# Integrate prediction of machine learning for single ACoA rupture risk: a multicenter retrospective analysis

**DOI:** 10.3389/fneur.2023.1126640

**Published:** 2023-10-18

**Authors:** Yang Li, Linchun Huan, Wenpeng Lu, Jian Li, Hongping Wang, Bangyue Wang, Yunfei Song, Chao Peng, Jiyue Wang, Xinyu Yang, Jiheng Hao

**Affiliations:** ^1^Department of Neurosurgery, Tianjin Medical University General Hospital, Tianjin, China; ^2^Department of Neurosurgery, Linyi People's Hospital of Shandong Province, Linyi, Shandong, China; ^3^Department of Neurosurgery, The First People's Hospital of Jining, Jining, Shandong, China; ^4^Department of Neurosurgery, Tangshan Workers Hospital, Tangshan, Hebei, China; ^5^Department of Neurosurgery, Liaocheng People's Hospital, Liaocheng, Shandong, China

**Keywords:** AcoA, machine learning, rupture risk factors, prediction model, web dynamic nomogram

## Abstract

**Background:**

Statistically, Anterior communicating aneurysm (ACoA) accounts for 30 to 35% of intracranial aneurysms. ACoA, once ruptured, will have an acute onset and cause severe neurological dysfunction and even death. Therefore, clinical analysis of risk factors related to ACoA and the establishment of prediction model are the benefits to the primary prevention of ACoA.

**Methods:**

Among 1,436 cases of single ACoA patients, we screened 1,325 valid cases, classified risk factors of 1,124 cases in the ruptured group and 201 cases in the unruptured group, and assessed the risk factors, respectively, and predicted the risk of single ACoA rupture by using the logistic regression and the machine learning.

**Results:**

In the ruptured group (84.8%) of 1,124 cases and the unruptured group (15.2%) of 201 cases, the multivariable logistic regression (MLR) model shows hemorrhagic stroke history (OR 95%CI, *p*:0.233 (0.120–0.454),<0.001) and the age stratification of 60–69 years (OR 95%CI, *p*:0.425 (0.271–0.668),<0.001) has a significant statistic difference. In the RandomForest (RF) model, hemorrhagic stroke history and age are the best predictive factors.

**Conclusion:**

We combined the analysis of MLR, RF, and PCA models to conclude that hemorrhagic stroke history and gender affect single ACoA rupture. The RF model with web dynamic nomogram, allows for real-time personalized analysis based on different patients’ conditions, which is a tremendous advantage for the primary prevention of single ACoA rupture.

**Clinical trial registration:**

https://www.chictr.org.cn/showproj.html?proj=178501.

## 1. Introduction

Intracranial aneurysm (IA), the leading cause of subarachnoid hemorrhage (SAH), is the third commonest cerebrovascular disease after the hypertensive hemorrhage and stroke. European Stroke Organisation (ESO) (2022) guidelines suggest that approximately 3% of the global normal adult population currently has an unruptured intracranial aneurysm (UIA) (1, 2). However, when an IA ruptures, it often causes the SAH, and the fundamental cause of approximately 85% of SAH is a ruptured intracranial aneurysm (RIA). SAH is a life-threatening subtype of stroke with a high morbidity and mortality rate (2, 3). It is estimated that approximately one-third of patients die and another third remain dependent on others for daily activities of living. Its high incidence, wide range, and poor prognostic outcome make it a cerebrovascular disease that seriously endangers human health, so it is worth noting how to more scientifically and effectively prevent and treat the rupture of IAs.

As the anterior communicating artery (AComm) of the anterior circulation of the cerebrovascular system, it is located lateral to the optic cross and is an essential part of the Willis loop at the base of the brain, and is also a common site for IAs, with the incidence of anterior communicating artery aneurysms (ACoA) accounting for approximately 30–35% of overall intracranial aneurysms. Clinical observation studies by Japanese scholars8 (including the data of aneurysm location) have shown that the frequency of ACoA rupture is relatively high, with rupture rates ranging from 24 to 43%. Relevant studies at home and abroad mainly investigate and discuss the risk factors, hemodynamics, and anatomy of ACoA (4–10), but rarely explore and analyze whether ACoA is the main risk factor to cause the rupture of arterial aneurysm with a large case data volume (≥1,000 cases). Considering these results, more recent studies has focused on the primary prevention of rupture of single ACoA by identifying and addressing modifiable risk factors.

Most risk predictions of IA rupture use traditional regression models, which assume that the predictor variables are linearly correlated with the risk of rupture. Such a model, therefore, cannot effectively exclude covariance and hidden relationships between predictor variables. We consider adopting statistical methods that can perform multidimensional calculations and detect effects between predictor variables so that the risk of rupture of IA can be predicted more accurately and more precise relationships between predictor variables and rupture risk can be explored.

The RandomForest (RF) model, a relatively new prediction model in machine learning. It performs well on test sets that introduce two randomities (random samples, random features) making RF less prone to overfitting. It can be trained fast and implemented to process large-scale datasets thanks to out-of-bag (OOB) data; in the training process, interactions between features can be detected, and the importance of features can be derived to provide a reference for the study. We hope that the inclusion of the RF model can improve the predictive ability of the prediction model and provide better guidance on the primary prevention of single ACoA.

## 2. Data and methods

### 2.1. Ethics statement

The Ethics Committee of the General Hospital of Tianjin Medical University approved the study protocol.

### 2.2. Data sources

The data for the 1,436 patients in this study were obtained from the Chinese Multi-Center Cerebral Aneurysm Database (CMAD), a database project of multicenter, prospective, observational studies set up by Department of Neurosurgery, General Hospital of Tianjin Medical University. CMAD was accepted as a partner registry by the Chinese Clinical Trial Registry (ChiCTR2100054014). It has 12 partner hospital units with data entry in 10 cities. The partner units in Tianjin, Hebei, Shandong and Shanxi provinces have considerable local visibility and authority in neurosurgery.

### 2.3. Data entry, exclusion and classification criteria

#### 2.3.1. Missing value processing

In 1436 cases, 88 cases have missing values. The missing values are as follows:

1. Medical history:
  a) hemorrhagic stroke history (10 cases).
  b) dyslipidemia (45 cases).
  c) diabetes mellitus (10 cases).
  d) hypertension (15 cases).
2. Habits:
  a) alcohol history (17 cases).
  b) tobacco history (7 cases).

The multiple missing values exist in some individual cases of the 88 cases. The missing rate is 6.13%. The missing values are caused by the physician not asking for or recording the relevant data during the patients’ visits. The missing values data are missing completely at random (MCAR) that the reason for the missing values was not related to any variable. However, the missing rate is low, so the remaining 1,348 cases with complete data are still representative. We took the direct approach of deleting the data to avoid the missing values affecting the overall complete data analysis (11).

#### 2.3.2. Selective bias treatment

It is well known that selection bias is a systematic error that is naturally occurring and not caused by the intervention of the researcher’s subjective consciousness or bias. When we study the association of predictor variables with the risk of single ACoA rupture, selective bias can produce results that over-or under-estimate the role of predictor variables (strength of association) (12). Therefore, we designed to continue the treatment regarding selective bias based on the 1,348 cases without the missing values. The basis for selective bias in this study is the higher incidence of SAH in ruptured aneurysms among intracranial aneurysms, and the fact that cases with a ruptured aneurysm were in hospital admissions due to first-time hemorrhagic stroke (including SAH), which hemorrhage caused only by ruptured aneurysms, recorded in the database as part of a recurrent aneurysm rupture, may have had some biasing effect on the results considering their history of hemorrhagic stroke may have been more associated with rupture. To avoid the influence of selective bias on the results of this study as far as possible, we excluded 23 cases (9 cases with cerebral hemorrhages, 3 cases with SAH, 5 cases with cerebral infarctions, and 6 cases with other cerebral hemorrhages) who have single ACoA.

#### 2.3.3. Predictor and outcome variables

We selected valid 1,325 cases after excluding the cases with missing values and selective bias, and classified them according to risk factors that may have some relevance for rupture. The categories are as follows.

1. Predictor variables.
  - Gender.
  - Age stratification-four strata:
    a) <50;
    b) 50–59;
    c) 60–69:
    d) ≥ 70.
  - Medical history.
    a) hypertension (systolic blood pressure ≥ 140 mmHg or diastolic blood pressure ≥ 90 mmHg/including the efficacy of antihypertensive drugs).
    b) diabetes mellitus (fasting blood glucose≥7 mmol/L or random postprandial blood glucose≥11.1 mmol/L).
    c) dyslipidemia (TC ≥ 6.2 mmol/L; LDL-C ≥ 4.1 mmol/L; TG ≥ 2.3 mmol/L; HDL-C < 1.0 mmol/L).
    d) ischemic stroke history.
    e) hemorrhagic stroke history.
    f) polycystic kidney disease.
  - Habits.
    a) tobacco history (regular, recent/non-smokers).
    b) alcohol history (recent, regular, abstainers/non-drinkers).
2. Outcome variables: ruptured or non-ruptured single ACoA.

### 2.4. Analysis methods

The *X^2^* test was first used to verify that the above predictor variables were significantly correlated with the outcome variables. Univariable logistic regression (LR) was used to explore which predictor variable correlated with the outcome variable initially. Afterward, the predictor variables that were strongly correlated with the outcome variable in LR were subjected to the multivariable logistic regression (MLR) to assess the level of influence of the predictor variables with strong correlations on whether the single ACoA ruptured (6, 13).

Secondly, MLR does not entirely rule out collinearity between predictor variables and in turn interferes with the conclusions about the level of influence of predictor variables on whether a single ACoA ruptures or not. Therefore, we used principal component analysis (PCA) to downscale the original predictor variables into a new set of several mutually uncorrelated synthetic variables, from which a few fewer sum variables could be taken out based on the practical needs to reflect as much information as possible about the original variables. PCA is to retain the new features that are most likely to reconstruct the original features, thus achieving the effect of dimensionality reduction of the data. The results of the final 1,325 cases were visualized in three-dimensions to aid the prediction (6, 14).

In contrast to the traditional MLR approach, RF comprehensively assesses various predictor variables. From there, we will extract the importance scores from the predictor variables and their significance, and visualize the importance weights of each predictor variable after ranking the predictor variables (6).

As for the results, a nomogram was used as a visualization tool to give it predictive modeling capability by setting up a training set and a test set. It used multiple clinical risk factors, assigned a score to each risk factor at each value level, then added up the individual scores to obtain a total score, and finally calculated the individual’s single ACoA rupture predictive probability by the function transformation relationship between the total score and the occurrence probability of a single ACoA rupture. The nomogram makes the results of the prediction model more readable and has higher use (13, 15, 16).

Finally, receiver operating characteristic (ROC) curve was used to evaluate the predictive ability of MLR and RF models. The sensitivity and specificity of each model in predicting the risk of a single ACoA rupture were obtained by graphically combining sensitivity and specificity and using the maximized Uden index to determine the cut-off point (7, 17).

### 2.5. Analytical tools

Data were analyzed by using the statistical software of R studio (2022-05-20, vision) and SPSS (26.0.0.0, x64 vision). The R package was used for PCA, RF model, ROC curves, and nomogram. SPSS was used for missing values processing, descriptive statistics, *X^2^* tests, LR and MLR. The nomogram was implemented in the form of a web dynamic nomogram, using the Shiny app^1^ to create an HTTP server that interacts with the web browser and a nomogram interface that interacts with the server to form a web dynamic nomogram.

## 3. Results

### 3.1. Descriptive statistics

Table 1 details the data based on the baseline characteristics of the ruptured and unruptured groups of the single ACoA. A total of 1,325 cases has completed data among which the ruptured group is 1,124 cases and the unruptured group is 201 cases. In the *X^2^* test, there was a statistically significant difference between the ruptured and unruptured groups in ages stratification (*p* < 0.001), hypertension (*p* = 0.005), diabetes mellitus (*p* = 0.001), dyslipidemia (*p* < 0.001), ischemic stroke history (*p* < 0.001) and hemorrhagic stroke history (*p* < 0.001), which means that the above predictor variables are related to a single ACoA rupture to some extent.

**Table 1:** Baseline characteristics of the ACoA study used to compare patients with ruptured ACoA and unruptured ACoA.

Characteristic	All (*n* = 1,325)	Ruptured (*n* = 1,124)	Unruptured (*n* = 201)	*p* value
*Gender*				
Male	696 (52.5%)	587 (52.2%)	109 (54.2%)	0.600
Female	629 (47.5%)	537 (47.8%)	92 (45.8%)	
*Ages*				
<50	360 (27.2%)	328 (29.2%)	32 (15.9%)	<0.001
50–59	423 (31.9%)	361 (32.1%)	62 (30.8%)	
60–69	388 (29.3%)	304 (27.0%)	84 (41.8%)	
≥70	154 (11.6%)	131 (11.7%)	23 (11.4%)	
*Medical history*				
Hypertension (yes)	716 (54.0%)	589 (52.4%)	127 (63.2%)	0.005
Hypertension (no)	609 (46.0%)	535 (47.6%)	74 (36.8%)	
Diabetes mellitus (yes)	74 (5.6%)	53 (4.7%)	21 (10.4%)	0.001
Diabetes mellitus (no)	1,251 (94.4%)	1,071 (95.3%)	180 (89.6%)	
Dyslipidemia (yes)	18 (1.4%)	10 (0.9%)	8 (4.0%)	<0.001
Dyslipidemia (no)	1,307 (98.6%)	1,114 (99.1%)	193 (96.0%)	
Polycystic kidneys (yes)	2 (0.2%)	1 (0.5%)	1 (0.1%)	0.169
Polycystic kidneys (no)	1,323 (99.8%)	1,123 (99.9%)	200 (99.5%)	
Ischemic stroke history (yes)	63 (4.8%)	42 (3.7%)	21 (10.4%)	<0.001
Ischemic stroke history (no)	1,262 (95.2%)	1,082 (96.3%)	180 (89.6%)	
Hemorrhagic stroke history (yes)	40 (3.0%)	22 (2.0%)	18 (9.0%)	<0.001
Hemorrhagic stroke history (no)	1,285 (97.0%)	1,102 (98.0%)	183 (91.0%)	
*Habits*				
Tobacco history (yes)	284 (21.4%)	241 (21.4%)	43 (21.4%)	0.988
Tobacco history (no)	1,041 (78.6%)	883 (78.6%)	158 (78.6%)	
Alcohol history (yes)	211 (15.9%)	186 (16.5%)	25 (12.4%)	0.142
Alcohol history (no)	1,114 (84.1%)	938 (83.5%)	176 (87.6%)	

All Values are expressed as numbers (%).

Student’s *χ*^2^ test were applied to see if there is a difference between patients with Ruptured group or Unruptured group.

### 3.2. LR and MLR models

In Table 2, LR utilizes odds ratio (OR) to explain the correlation of predictor variables for the rupture of the single ACoA. Gender, tobacco history, and the history of polycystic kidney disease have no significant statistical significance, and these three predictor variables will not be included in the MLR. By comparison, the ages of patients have a certain influence on single ACoA rupture. Compared with patients younger than 50 years old, the age of patients aged 50 years and above is judged to be a protective factor (ages stratification, OR and *p*-values: <50 years, reference; 50–59 years, 0.568, 0.014; 60–69 years, 0.353,<0.001; ≥70 years, 0.556, 0.044). In LR, except for the history of polycystic kidney disease which is not statistically significant, all the other disease histories which influence the risk of single ACoA rupture are considered as protective factors.

**Table 2:** OR calculation for univariable logistic regression (LR) to explain ACoA risk factors of the rupture.

Explanatory variables	OR
*Gender (male)*	
Female	1.084 (0.802–1.465, *p* = 0.600)
*Ages(<50)*	
50–59	0.568 (0.361–0.893, *p* = 0.014)
60–69	0.353 (0.228–0.546, *p* < 0.001)
≥70	0.556 (0.313–0.985, *p* = 0.044)
*Medical history*	
Hypertension (yes)	0.641 (0.471–0.874, *p* = 0.005)
Diabetes mellitus (yes)	0.424 (0.250–0.720, *p* = 0.001)
Dyslipidemia (yes)	0.217 (0.084–0.556, *p* = 0.001)
Polycystic kidneys (yes)	0.178 (0.011–2.859, *p* = 0.223)
Ischemic stroke history (yes)	0.333 (0.193–0.575, *p* < 0.001)
Hemorrhagic stroke history (yes)	0.203 (0.107–0.386, *p* < 0.001)
*Habits*	
Tobacco history (yes)	1.003 (0.696–1.446, *p* = 0.998)
Alcohol history (yes)	1.396 (0.892–2.184, *p* = 0.144)

Values are expressed as OR (95% CI, *p* value).

**Figure 1:**
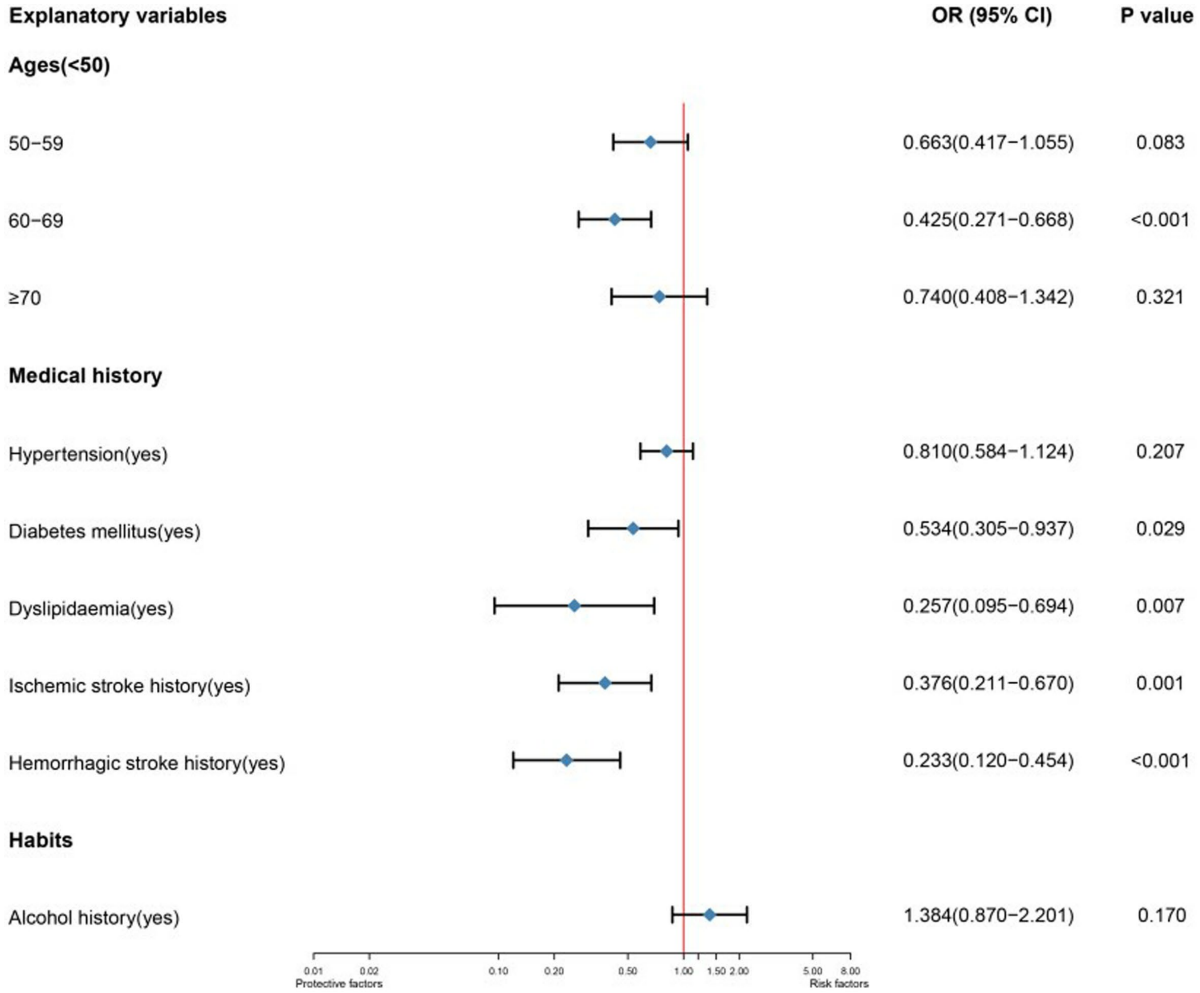
Forest plot of a multivariable logistic regression to explain single ACoA rupture. The model’s variables are represented depending on their labels. CI indicates confidence interval; OR, odds ratio.

As there were only ten predictor variables, we adjusted the selection of predictor variables that could be included in MLR and added the predictor variables with *p* < 0.15 in LR to MLR. Figure 1 is the forest plot of the MLR. In the MLR, all predictor variables in medical history are shown to be protective factors. Among those variables, hemorrhagic stroke history (OR 95%CI, *p*:0.233 (0.120–0.454),<0.001) has a very significant statistic difference, ischemic stroke history (OR 95%CI, *p*:0.376 (0.211–0.670),0.001), dyslipidemia (OR 95%CI, *p*:0.257 (0.095–0.694),0.007) and diabetes mellitus (OR 95%CI, *p*:0.534 (0.305–0.937),0.029) have statistic difference. In the age, the age stratification of 60 and 69 years as an independent protective factor (OR 95%CI, *p*:0.425 (0.271–0.668),<0.001) has a statistical difference.

### 3.3. RF model

**Figure 2:**
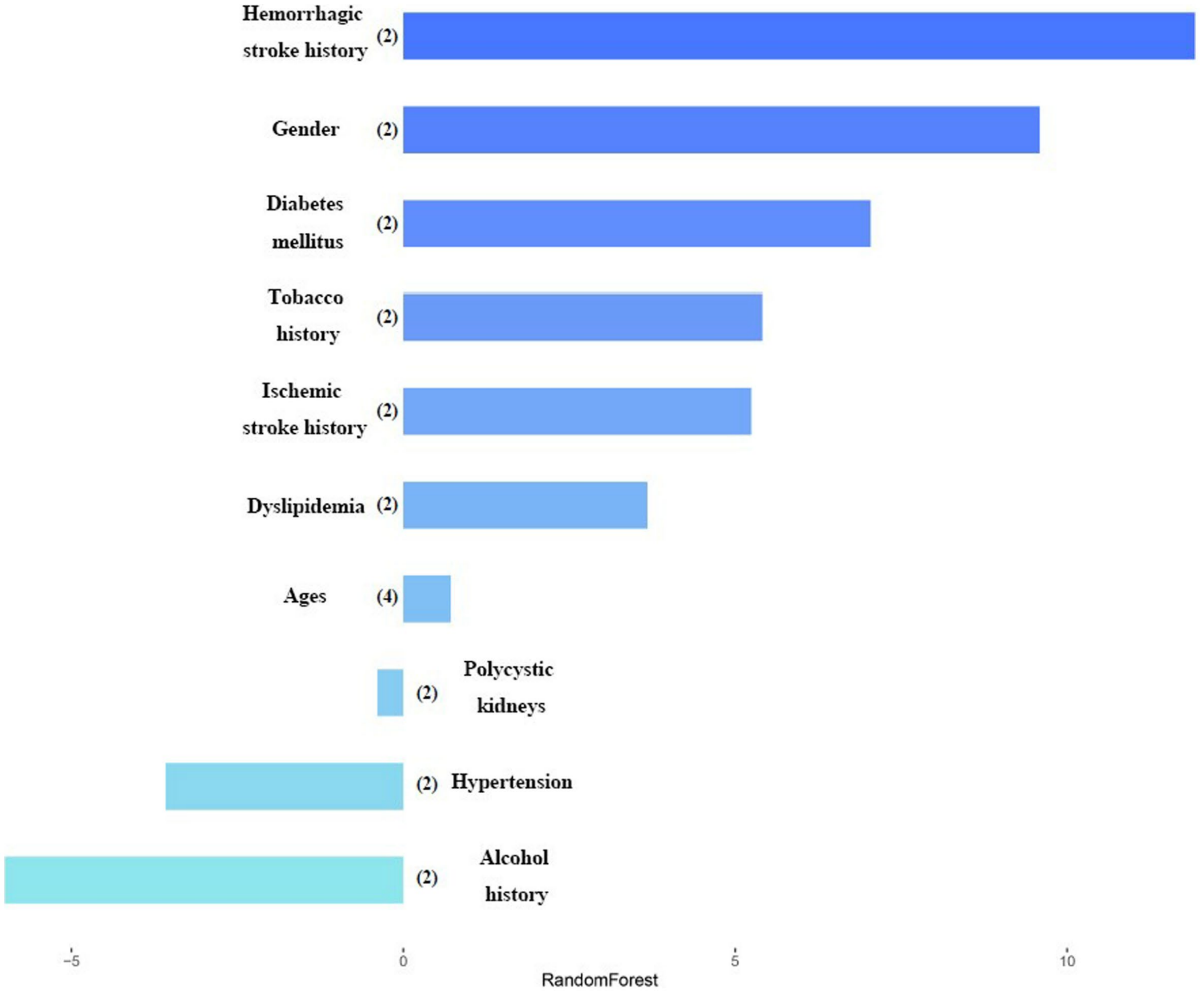
Variables importance in random forest model. After each variable name, the number of labels is indicated within the parentheses. Variables are classified by the most important impact in the random forest model.

Figure 2 shows the weight graph of each predictor variable under RF model. We set up 4 models with different seed numbers in RF model (0, 500, 1,000, 2000), among which the lowest Out Of Bag Error (OOB error) is 15.58%. The model with the lowest out-of-bag error was selected and iteratively adjusted parameters to select the one with the best classification effect. After ten times 10-fold cross-validation, the mean value of the error rate of the OOB-based model was obtained by improving the parameters, where the adjusted OOB was 14.41%. This method effectively controlled the error rate. After that, the importance and significance of each predictor variable were extracted and sorted and visualized to obtain the weight graph of the importance of each predictor variable on whether it leads to the single ACoA rupture. It been seen from the plot that hemorrhagic stroke history, gender, and diabetes mellitus ranked the top three risk factors for rupture, respectively, in RF model.

### 3.4. PCA

In order to avoid collinearity among predictor variables, PCA was adopted to add all predictor variables of cases’ data into the PC without potential mutual influence. PCA was used to describe the distribution of cases and figure out the tendency of the influence of predictor variables on single ACoA cases.

**Figure 3:**
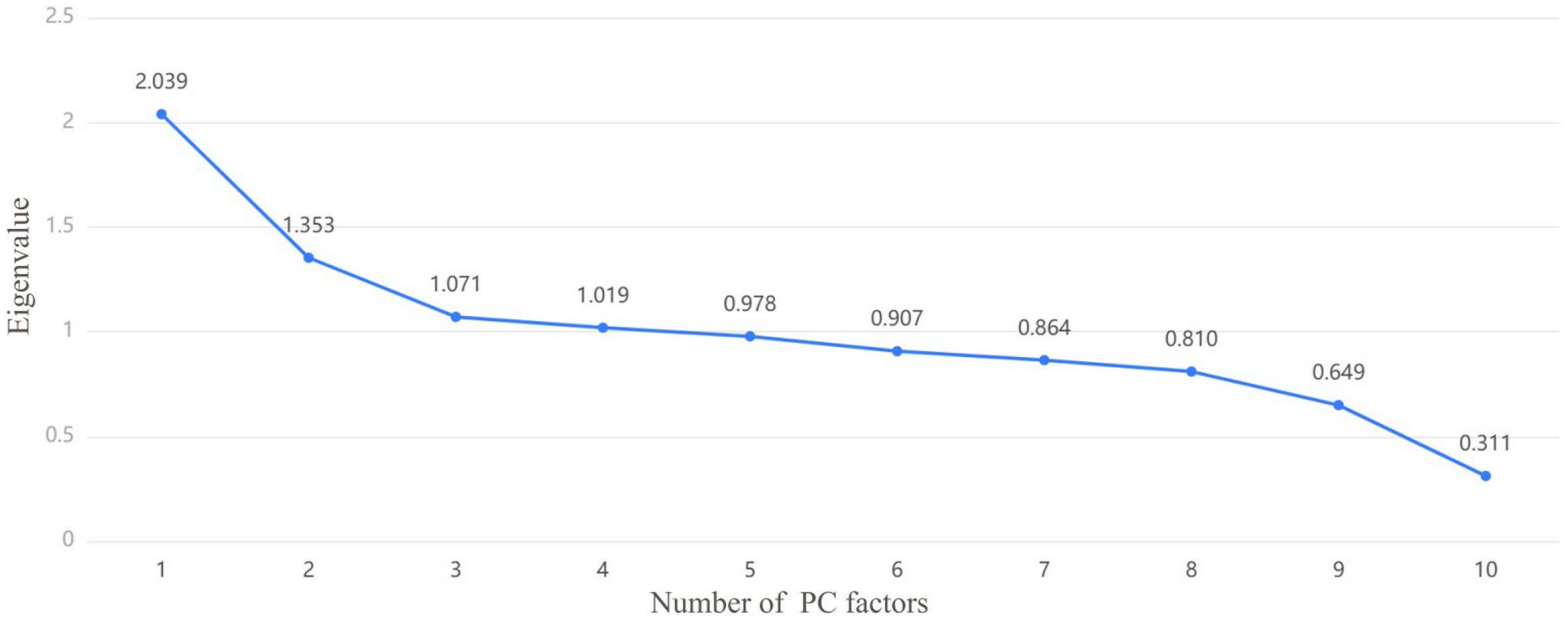
The screen test determines the number of principal components (PC) selected based on the slope of the decline in eigenvalues, which can be combined with the variance interpretation lable to determine or adjust the number of PC factors. Each PC is a point, and the number of PCs extracted is determined by the slope.

Firstly, KMO and Bartlett tests were conducted, KMO = 0.62, Bartlett *p* < 0.001, showing statistical significance. The correlation between the predictor variables was found, and PCA was effective and the degree was generally suitable. Figure 3 is the scree plot. The first three PC factors show a steeper trend in slope, indicating that they have large differences and obvious characteristics; the slope between the third PC factor and the fourth and subsequent PC factors is gentle and their eigenvalues are similar. Therefore, it can be concluded that the first three PCs are treated as the relatively important PCs in this study.

**Figure 4:**
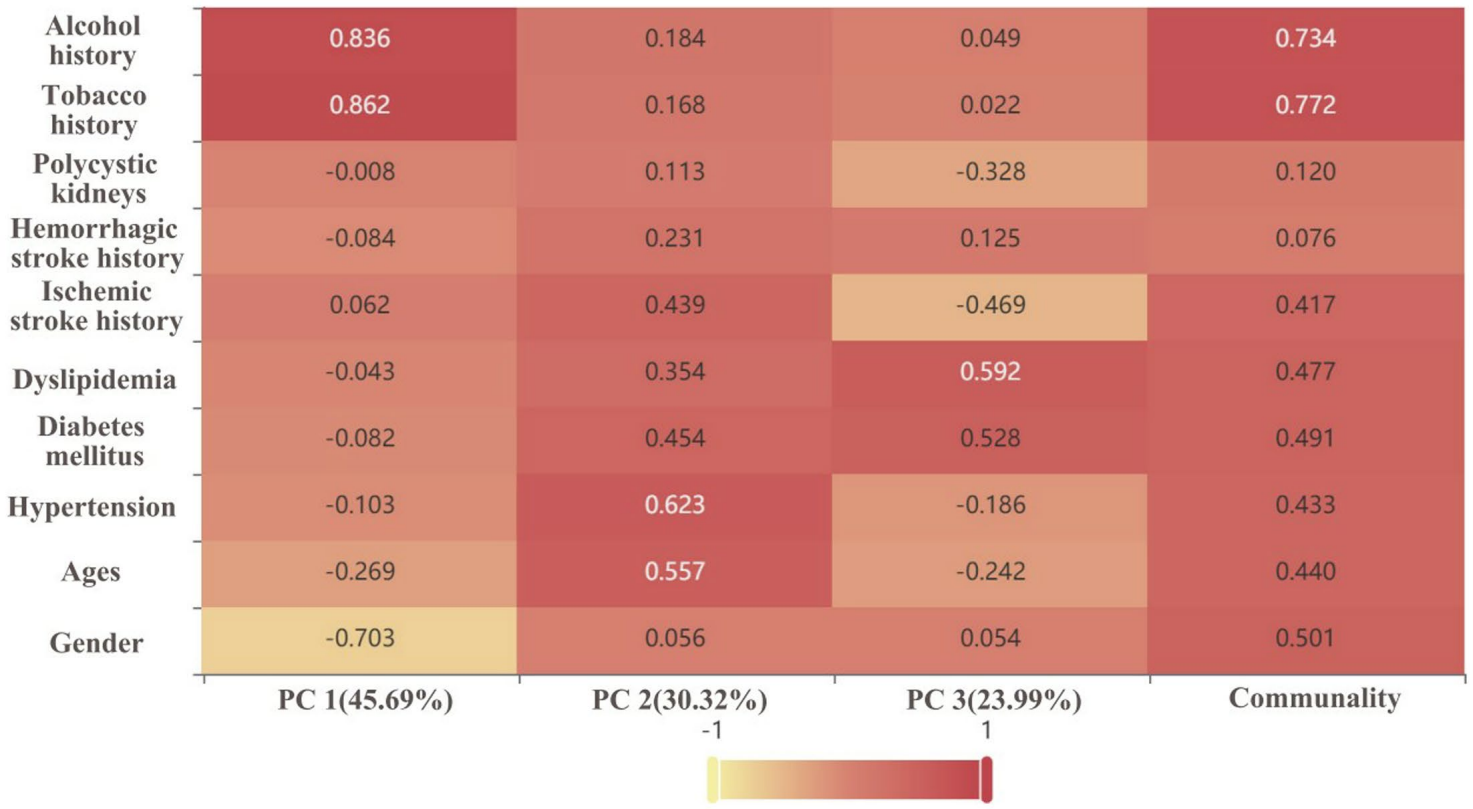
Factor loading matrix heat map analyses the significance of the hidden variables in each principal component (PC). The darker the color/the closer the value to 1, and the higher the significance of the hidden variable. The percentage is the weight of the PC.

**Figure 5:**
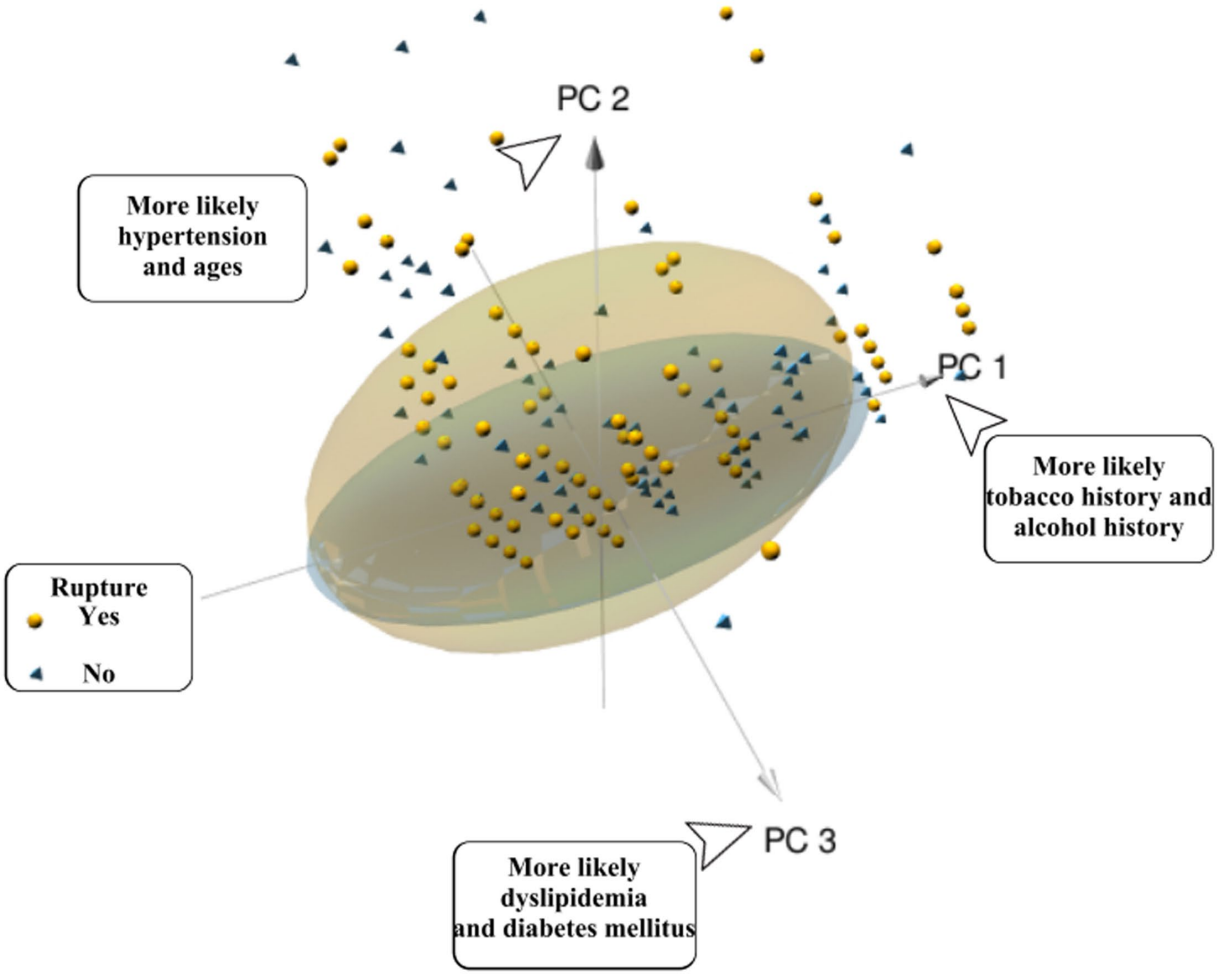
Patients with a ruptured single ACOA (yellow spheres) or an unruptured single ACOA (blue vertebrae) are represented the plot. The position relative to each PC-axis gives some patients’ characteristics. Yellow spheres represent the patients in the ruptured group; yellow ellipses represent 95% CI of the ruptured group. Blue pyramids represent patients from the unruptured group; blue ellipses represent 95% CI of the unruptured group.

Through the ratio of hidden variables, we defined which predictor variables each of the three PC factors represented, and made a factor loading matrix heat map (Figure 4). Tobacco history (0.862) and alcohol history (0.836) were the main hidden variables in PC1 (45.69% weight ratio). Hypertension (0.623) and age (0.557) were the main hidden variables in PC2 (30.32% weight ratio), while diabetes mellitus (0.454), ischemic stroke history (0.439), dyslipidemia, hemorrhagic stroke history (0.231) were secondary hidden variables. Figure 5 is a three-dimensional diagram of PCA.

## 4. Discussion

**Figure 6:**
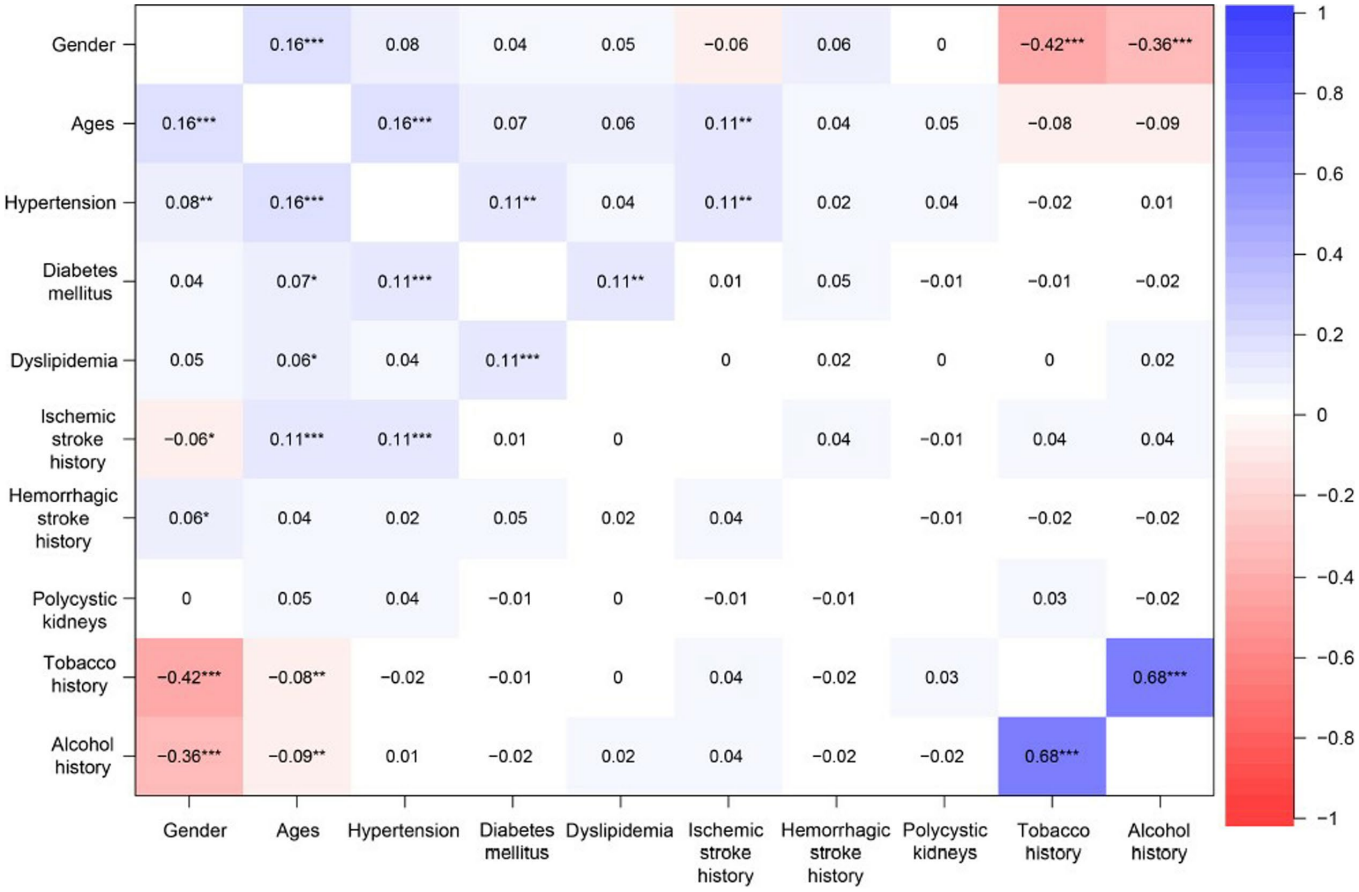
The heatmap of relevance analysis shows the links between the predictor variables. Positive values represent positive correlations between predictor variables; negative values represent negative correlations between predictor variables; the magnitude of the value represents the strength of the correlation between predictor variables. * indicates significant difference (*p* < 0.05):** indicates significant difference (*p* < 0.01):*** indicates significant difference (*p* < 0.001).

In this study, we developed a predictive model for the risk of single ACoA rupture in CMAD using a traditional MLR model and the popular machine learning-RF model, and it is validated through the ROC. Secondly, we used PCA to perform a somewhat complementary role in predicting the prediction model. We expressed the prediction model results in a form of a web dynamic nomogram (Figure 6).

### 4.1. Analysis of prediction model results

In 1325 cases, most of the ruptured groups were distributed in the positive axis of PC2 in PCA, and scattered in the directions of PC1 and PC3. We judged that rupture of single ACoA was more correlated with hypertension, age, diabetes mellitus, ischemic stroke history, dyslipidemia, and hemorrhagic stroke history. However, PCA could not express specific values by the correlation of predictor variables with the cause of rupture in three-dimensional diagram. In contrast, the ruptured group was more distributed in the 95% CI ellipse than the unruptured group, which may be related to the fact that the sample size of the ruptured group was approximately five times larger than that of the unruptured group. It was impossible to determine whether this distribution was due to the high confidence level of the ruptured group (18) (PCA is only used as an auxiliary statistical method to observe the likelihood of interactions between variables and does not affect the predicted outcome.)

In MLR, diabetes mellitus, dyslipidemia (19), ischemic stroke history, hemorrhagic stroke history and age stratification for those aged 60–69 years were all independent protective factors. This is consistent with the results of Olivia Rousseau and his colleagues working on the ICAN project study in the UK^6^.

In RF model, hemorrhagic stroke history and gender were highly weighted as predictive factors of single ACoA rupture and were the two most important factors influencing single ACoA rupture, which is consistent with the epidemiological conditions. A review article on stroke in China shows (20–24) that stroke is the second leading cause of death not only in the world but also in China which accounts for one-fifth of the world’s population. Nationally, the prevalence of stroke is highest in northern China, and the prevalence of stroke increases rapidly with age (25). Patients with stroke account for 2–5% of adults and result in higher disability-adjusted life years than all other diseases. In this study, single ACoA as a type of IA, and a history of hemorrhagic stroke in patients with single ACoA in RF are important factors influencing the rupture of single ACoA. In the study by Zhen Xu and his colleagues, SAH caused by aneurysm rupture, which is a type of hemorrhagic stroke, had a higher prevalence of stroke in northern China, and the correlation between SAH and rupture of IA was higher in hemorrhagic stroke. This is similar to the idea of the study by Zhen Xu and colleagues (21).

The gender factor is the second important influence factor in RF, and in RF model of ICAN, it has some importance for IA rupture (the gender factor ranks the sixth out of 27 predictor variables) (6). In a Meta-analysis of individual patient data by Charlotte C.M. Zuurbier, MD, Ph.D. (26), it was explained that the risk of rupture in UIAs is generally higher in female than in male due to referring to the gender factor, which may be affected by the hormonal and reproductive factors specific to female (27). For example, these factors, such as genetic factors on the X chromosome, gender-specific effects of environmental risk factors, tobacco, or other unknown clinical factors, occur more frequently or have a more significant impact in female than in male, explain the possibility that gender difference exists (26, 27).

A history of polycystic kidney disease (containing ADPKD, one of the common factors causing death due to SAH in IAs) had a lower level of impact in the RF model of this study. The most common site of an aneurysm in patients with common ADPKD is the middle cerebral artery and the most common site of aneurysm rupture in patients with ADPKD is the AComm (28, 29). However, the polycystic kidney disease has less impact on a single ACoA rupture in RF model. The reason of judgement is that there are only 2 cases (0.1%) with a history of polycystic kidney disease in this study, which significantly reduced the validity and predictive accuracy of this predictor variable.

### 4.2. Comparison of prediction model accuracy

We use the ROC curves to conclude the accuracy of each prediction model.

**Figure 7:**
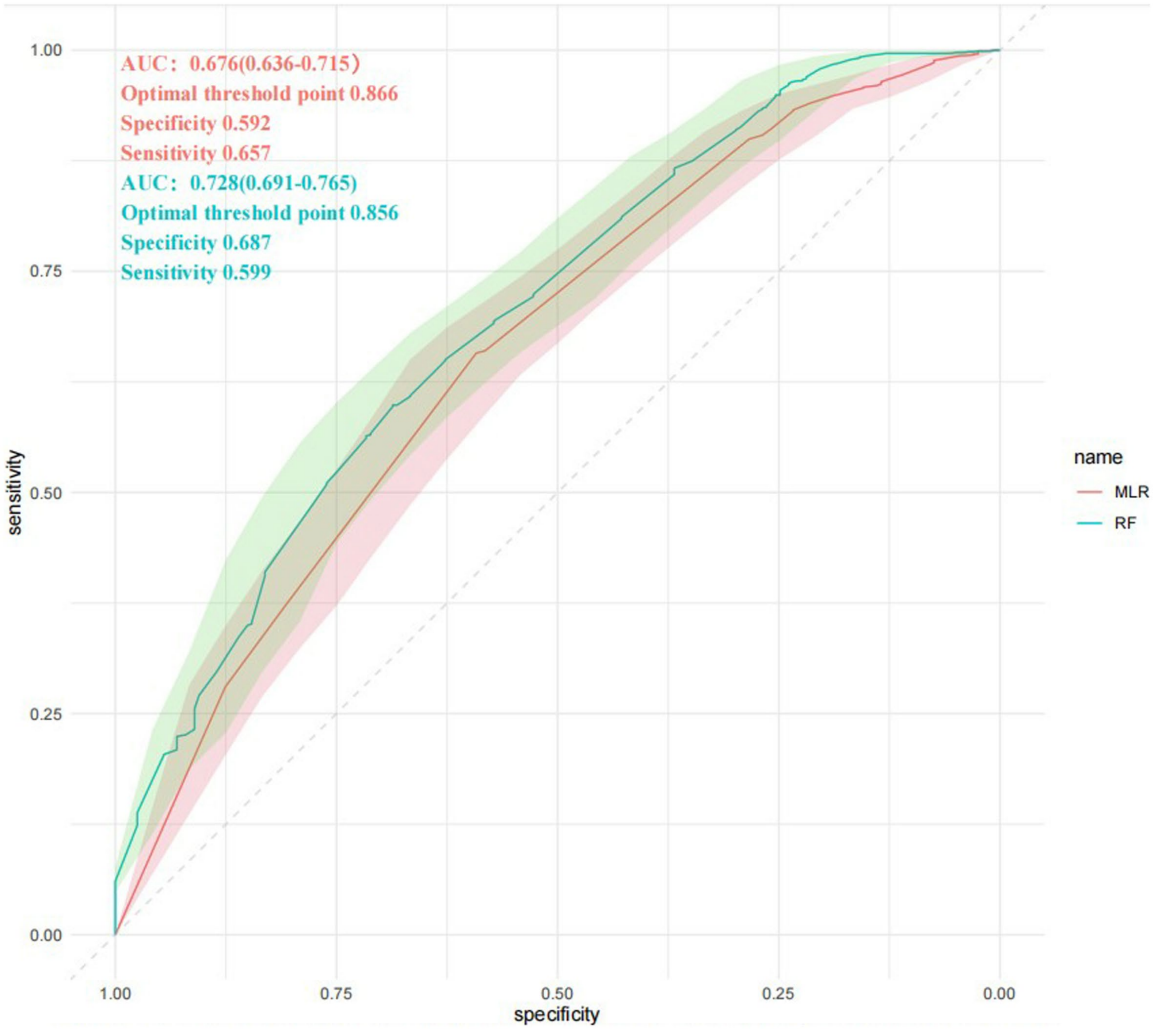
ROC curves comparison. RF, Random Forest model; MLR, multivariable logistic regression model. The AUC for each method is given by mean and SD interval. ROC curves are intersected several times. AUC, area under the curve; ROC, receiver operating characteristic.

Figure 7 is the ROC curves for MLR and RF model, comparing the predictive abilities of the two prediction models. The abilities are reflected in the prediction of whether the predictor variables cause a single ACoA rupture. The area under the curve or AUC in MLR model is 0.662 (95%CI: 0.621–0.703) and in RF model is 0.700 (95% CI:0.660–0.739). The optimal threshold point for ROC of MLR was 0.866, specificity was 0.59, and sensitivity was 0.657; the optimal threshold point for ROC of RF was 0.856, specificity was 0.68, and sensitivity was 0.599. It can show that RF model has a relatively higher accuracy of predictive ability than MLR model. Unlike the traditional MLR, the RF model has some advantages in significant sample size analysis that it does not need to consider the influence between predictor variables and to deal with missing values of variables, which are judged to be the reasons for the high accuracy of RF.

### 4.3. Visualization of web pages clinical prediction model

**Figure 8:**
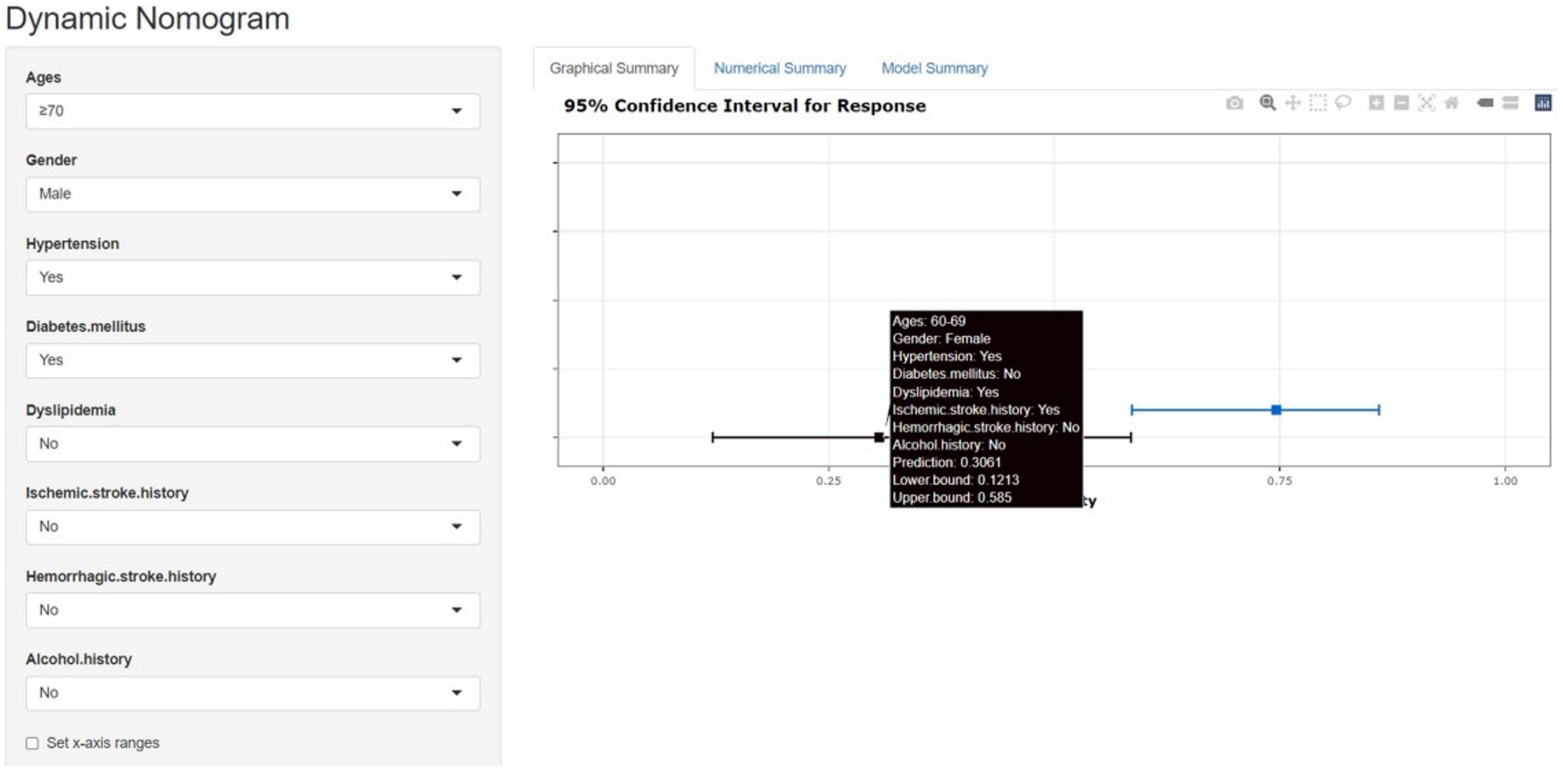
The nomogram model and the dynamic nomogram model predicts the rupture risk of single ACOA, based on ages, gender, hypertension, diabetes mellitus, dyslipidemia, ischemic stroke history, hemorrhagic stroke history and alcohol history. The dynamic nomogram model shows the single ACOA ruptured rate by the “Prediction” function (https://cmad.shinyapps.io/DynNomapp/).

Figure 8 is a web dynamic nomogram which divided 1,325 cases into a ratio of 3:1 according to the training set and the validation set, and controlled the predictor variables to be validated within the training set, and analyzed the multi-factor logistic regression in the training set and developed the nomogram of the risk of single ACoA rupture of the clinical prediction.

This study aimed to develop a prediction model for the risk of rupture of a single ACoA suitable for the normal population, i.e., patients with no incidence of the disease were predicted for the risk factors associated with ACoA through the initial prediction by establishing LR and RF prediction models for the collected cases, and a web dynamic nomogram was used, which allowed the input of relevant data from the uninfected population or the normal population to predict in real-time (13, 30).

As only one patient had polycystic kidney disease in this study, the case is not specific; tobacco history was not significant in either LR or MLR. These two predictor variables, therefore, were not included in the nomogram.^2^ The web dynamic nomogram allows the patient’s profile to be filled in according to the corresponding options of risk factor classification in the webpage, and figures out the probability of their risk of single ACoA rupture in real-time, which has more benefits than the traditional nomogram and is more favorable for the primary prognosis of single ACoA (Instructions on how to use the web dynamic nomogram are provided in the Supplementary materials).

## 5. Summary

We made a comprehensive judgment based on MLR, RF and PCA that hemorrhagic stroke history and gender have a certain influence on single ACoA rupture. The RF model of machine learning is a stable model for predicting the risk of single ACoA rupture in extensive sample-size studies. The RF combining with web dynamic nomogram can conduct real-time personalized analysis based on different patients’ conditions, which has greater advantages for primary prevention of single ACoA rupture.

## 6. Limitations

This study is based on CMAD and focuses on the risk factors related to single ACoA, and the differences between multiple and single incidence are not expounded. This study has geographical limitations for it only represents the incidences and ruptures of ACoA in northern China and does not discuss the cases and ethnic factors in different countries. In the future, we plan to add the analysis of various risk factors such as ethnicity, aneurysm size, anatomy, and hemodynamics and refine the classification criteria to improve prediction accuracy and provide better early warning for primary prevention of single ACoA.

## Data availability statement

The original contributions presented in the study are included in the article/Supplementary material, further inquiries can be directed to the corresponding authors.

## Ethics statement

The Ethics Committee of the General Hospital of Tianjin Medical University approved the study protocol. The data of patients in this study were obtained from the Chinese Multi-Center Cerebral Aneurysm Database (CMAD),^3^ a database project of multicenter, prospective, observational studies set up by Department of Neurosurgery, General Hospital of Tianjin Medical University. CMAD was accepted as a partner registry by the Chinese Clinical Trial Registry (ChiCTR2100054014).

## Author contributions

BW: conceptualization. BW and YL: methodology. YL: formal analysis and visualization. YL, LH, JL, WL, HW, BW, YS, CP, JW, XY, JH: writing – review and editing. JW, XY, JH: Project administration. XY and JH: supervision. All authors contributed to the article and approved the submitted version.

## Footnotes

^1^https://www.shinyapps.io/

^2^https://cmad.shinyapps.io/DynNomapp/

^3^http://www.cmadtj.com/
